# Annealing and thickness optimisation in CoZrNb and FeSiBNbCu thin films for low-frequency magnetic flux concentrators

**DOI:** 10.1016/j.jmmm.2025.173621

**Published:** 2025-11-04

**Authors:** H. Minh Cao, Benjamin J. Brown, Vineetha S. Bheemarasetty, Gang Xiao

**Affiliations:** Department of Physics, Brown University, Providence, RI 02912, USA

**Keywords:** CoZrNb, FeSiBNbCu, Magnetic flux concentrator, Low-frequency, Soft magnetic material, Annealing, Amorphous, Nanocrystalline, Thin films

## Abstract

To develop high-performance soft magnetic flux concentrator (MFC) materials, which are crucial for amplifying magnetic fields through the enhancement of local magnetic flux density, two ferromagnetic alloys, Co_88_Zr_4_Nb_8_ (CZN) and Fe_73.5_Si_15.5_B_7_Nb_3_Cu_1_ (Finemet^®^), were investigated. Thin films with thicknesses ranging from 100 to 1200 nm were deposited by magnetron sputtering and annealed at various temperatures. CZN demonstrated optimal soft magnetic properties in its as-grown amorphous configuration. In contrast, annealing significantly improved Finemet’s performance between 200°C and 500°C, where its nanocrystalline structure yielded ultra- soft behaviour. Additionally, thickness dependence revealed distinct trends in magnetic behaviour for both materials. For CZN, magnetic softness was best achieved at a larger thickness (1200 nm), while for Finemet, an optimum was observed at an intermediate thickness (800 nm). Thus, CZN is suited for cases where annealing is impractical, such as CMOS-compatible or MEMS-integrated sensors, as its properties are optimised without further processing, particularly in thicker films. Finemet, however, requires controlled annealing to obtain soft magnetic behaviour, making it suitable for applications that demand maximum permeability and can tolerate higher temperatures. Both materials demonstrate excellent potential for MFC integration in magnetic sensing and spintronic platforms. The optimal choice, however, depends on the thermal budget, processing conditions, and the performance metrics prioritised in the target application.

## 1. Introduction

Magnetic flux concentrators (MFCs) are passive components made from soft magnetic materials, engineered to locally amplify external magnetic fields. This magnetic softness arises from a combination of material properties and structural design such as the geometry, size, and internal domain configuration, which together enable efficient flux guidance and amplification [1–4].

While many bulk alloys exhibit outstanding magnetic softness characterised by high permeability and ultra-low coercivity, this performance often degrades in thin films due to enhanced magnetic anisotropy and stress effects [5–7]. Within this context, we focus on two systems that represent distinct, well-studied pathways to softness. First is the amorphous Co-based alloy Co_88_Zr_4_Nb_8_ (CZN), which can achieve very low coercivity without thermal treatment when deposited under ambient conditions, owing to random anisotropy averaging in the fully amorphous state and relatively small magnetostriction [8–10]. Second is the nanocrystalline Fe-based alloy Fe_73.5_Si_15.5_B_7_Nb_3_Cu_1_ (Finemet^®^), which requires a controlled annealing sequence to precipitate ultrafine *α*-Fe(Si) grains in an amorphous matrix, a microstructure known to deliver extremely high permeability and very low coercivity through exchange averaging [11–13]. When properly synthesised and processed, these alloys display excellent soft magnetic properties including high saturation magnetisation (*M*_s_), low remanence (*M*_r_), large magnetic permeability (*μ*), minimal differential anisotropy field (${Η}_{\text{k}}^{\text{diff}}$), and low coercivity (*H*_c_).

This pair offers a deliberate contrast that is directly relevant to thin-film MFCs. CZN is attractive when a low-temperature, as-deposited process is required, for example in post-CMOS or MEMS sensors where the thermal budget is limited [14–16]. Finemet is appealing when a brief thermal step is permissible, since the nanocrystalline state can provide higher permeability, very small anisotropy fields, and excellent reversibility [17–19]. In other words, CZN offers process compatibility and immediate softness without annealing, whereas Finemet offers ultimate magnetic performance after a short, well-controlled heat treatment. Comparing them side by side clarifies the trade-off between fabrication constraints and peak soft-magnetic metrics that device designers must consider.

In this study, we systematically investigate the magnetic softness of CZN and Finemet thin films, focusing on how their properties depend on annealing temperature and film thickness. We control sputtering and post-processing conditions to suppress unwanted crystalline phases and to minimise internal stress, such that *H* be compared directly. By examining the effects of thermal treatment and thickness scaling on these key parameters, we provide practical guidance for selecting material and process windows for integration into advanced solid-state magnetic sensors and spintronic devices [20].

## 2. Experimental procedures

High-purity (99.9%) sputtering targets of Co_88_Zr_4_Nb_8_ and Fe_73.5_Si_15.5_B_7_Nb_3_Cu_1_ were commercially obtained. Single-layer films were deposited at room temperature onto thermally oxidised silicon wafers using high-vacuum magnetron sputtering, with substrates mounted on self-rotating holders to promote uniform growth. Once the base pressure reached 3 × 10^−8^ Torr, ultra-high-purity Ar gas was introduced to ignite and sustain the plasma. CZN was grown under 65 W dc power at 1.75 mTorr, yielding a deposition rate of 2.76 Å/s. Finemet required 100 W dc at 4.00 mTorr, corresponding to 3.46 Å/s. Thin films with thicknesses of 100 nm, 400 nm, 800 nm, and 1200 nm were fabricated for both materials.

Substrate patterning was carried out using standard photolithography with a maskless aligner (Heidelberg Instruments MLA 150) to define 2 mm-diameter circles for magnetic characterisation. A positive photoresist mask, prebaked at 120 °C, was used for lift-off. Afterwards, these circles were measured using a vibrating sample magnetometer (VSM, MicroSense^®^ EZ7), which also enabled in-situ annealing in an Ar-rich environment. To assess magnetic anisotropy, hysteresis loops were measured along radial and tangential in-plane directions with respect to the centre of the substrates, corresponding to the deposition-induced symmetry from the rotating holders.

Samples were annealed in-plane under zero magnetic field with industrial grade argon flowing at 12 SCFH from the start of heating through complete cool-down. Sequential target temperatures from 21°C (room-temperature) to 550°C were applied with a continuous ramp of 75°C/minute. Each setpoint was held for 10 minutes, after which the sample was allowed to cool naturally in flowing Ar with a measured rate of 52°C/minute to ambient conditions. At this point, the gas flow was shut off and magnetic hysteresis curves were measured. No mechanical stress was applied during this process. The 10-minute dwell time minimised the thermal budget while still achieving the required soft magnetic response. This choice is confirmed later on to be sufficient for Finemet at 500°C.

From the resulting *M–H* loops, *M*_s_, *M*_r_, and *H*_c_ were extracted. Magnetic permeability was calculated following the expression $1+4\pi \frac{dM}{dH}$ , and *H*^diff^ was determined from the difference in saturation fields measured along the radial and tangential directions [5,21]. Due to the circular geometry and thicknesses of the samples, the shape demagnetisation factor is negligible [22,23], ensuring that the extracted magnetic parameters reflect intrinsic material properties rather than geometric effects.

X-ray diffraction (XRD, Bruker D8 Discover X-ray Diffractometer) with Cu-K*α* radiation (*λ* = 1.5418 Å) was used to characterise the crystalline structure of thin films as they evolve after each step of the annealing procedure. Roughness measurements over an area of 1 μm^2^ were performed by atomic force microscopy (AFM, Asylum MFP-3D Origin) to investigate the influence of surface roughness on both materials.

## 3. Results and discussions

To evaluate the magnetic softness of the films, we first focus on two key parameters: *M*_r_ and *H*_c_. These quantities directly influence energy loss due to hysteresis [24], which can be approximated by

(1) $$W_{h}=\oint HdM\approx 16\pi H_{c}M_{r}.$$

While low hysteresis loss (*W*_h_) is essential in high-frequency and saturation-driven applications, low-field and low-frequency magnetic sensors place greater emphasis on minimising coercivity *H*_c_. This helps maintain magnetic reversibility and suppress memory effects.

For MFC applications, even small coercive forces can introduce distortion into the amplified magnetic signal. As a result, assessing flux-guiding efficiency requires decoupling soft magnetic behaviour from any coercive or remanent interference. Only when coercivity is minimised do permeability and anisotropy become a meaningful metric for optimisation. Hence, in this study, we first identify samples with minimal hysteresis based on *H*_c_ and *M*_r_ before deriving *μ* and ${Η}_{\text{k}}^{\text{diff}}$).

### 3.1. Dependence of soft magnetic properties on annealing temperature

**Fig. 1.**
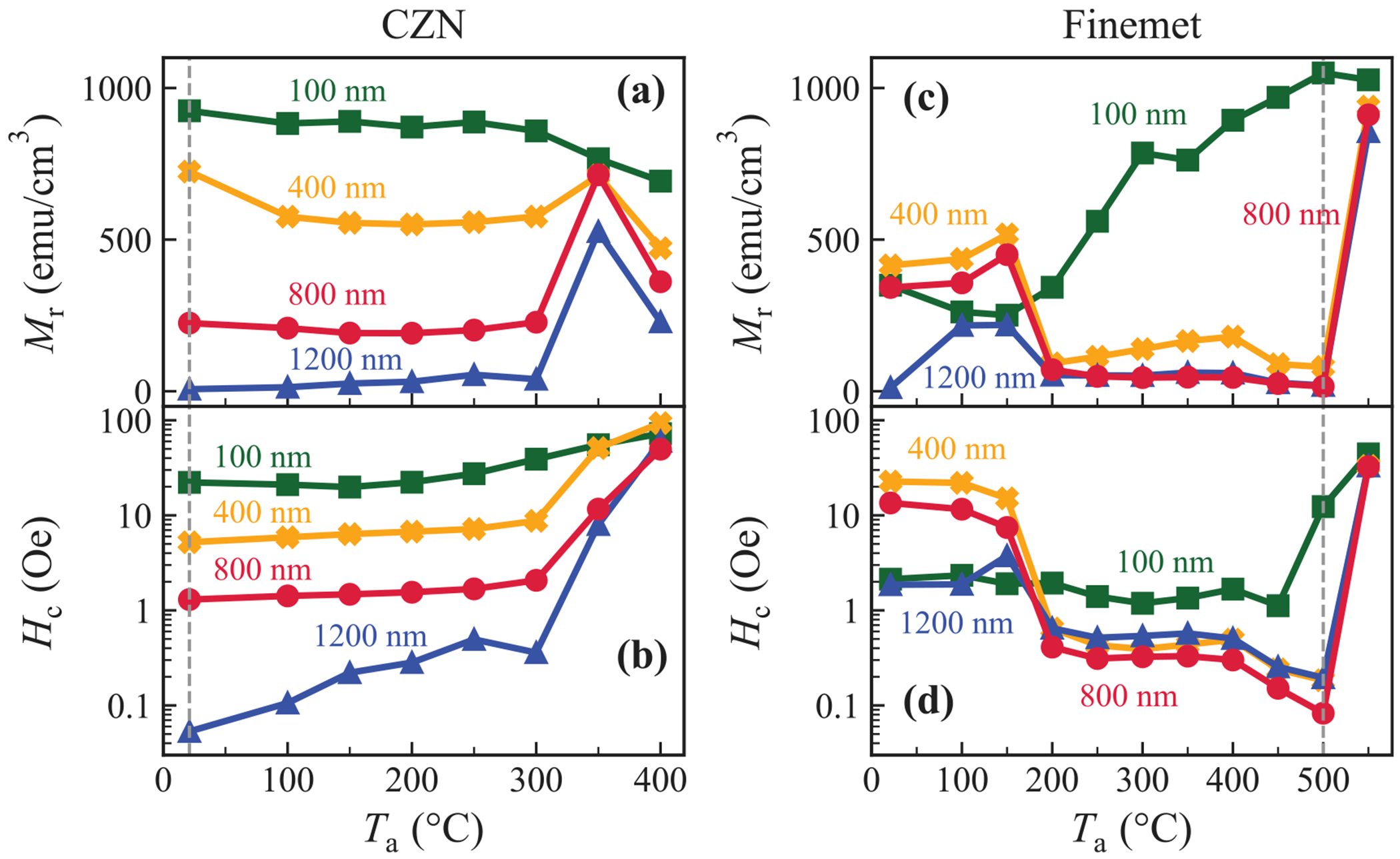
Remanent magnetisation (*M*_r_) and coercive field (*H*_c_) as a function of annealing temperature for (a,b) Co_88_Zr_4_Nb_8_ (CZN) and (c,d) Fe_73.5_Si_15.5_B_7_Nb_3_Cu_1_ (Finemet) thin films at varying thicknesses (100–1200 nm). Vertical dashed lines indicate key transitions in samples’ behaviour to optimal magnetic softness.

**Fig. 2.**
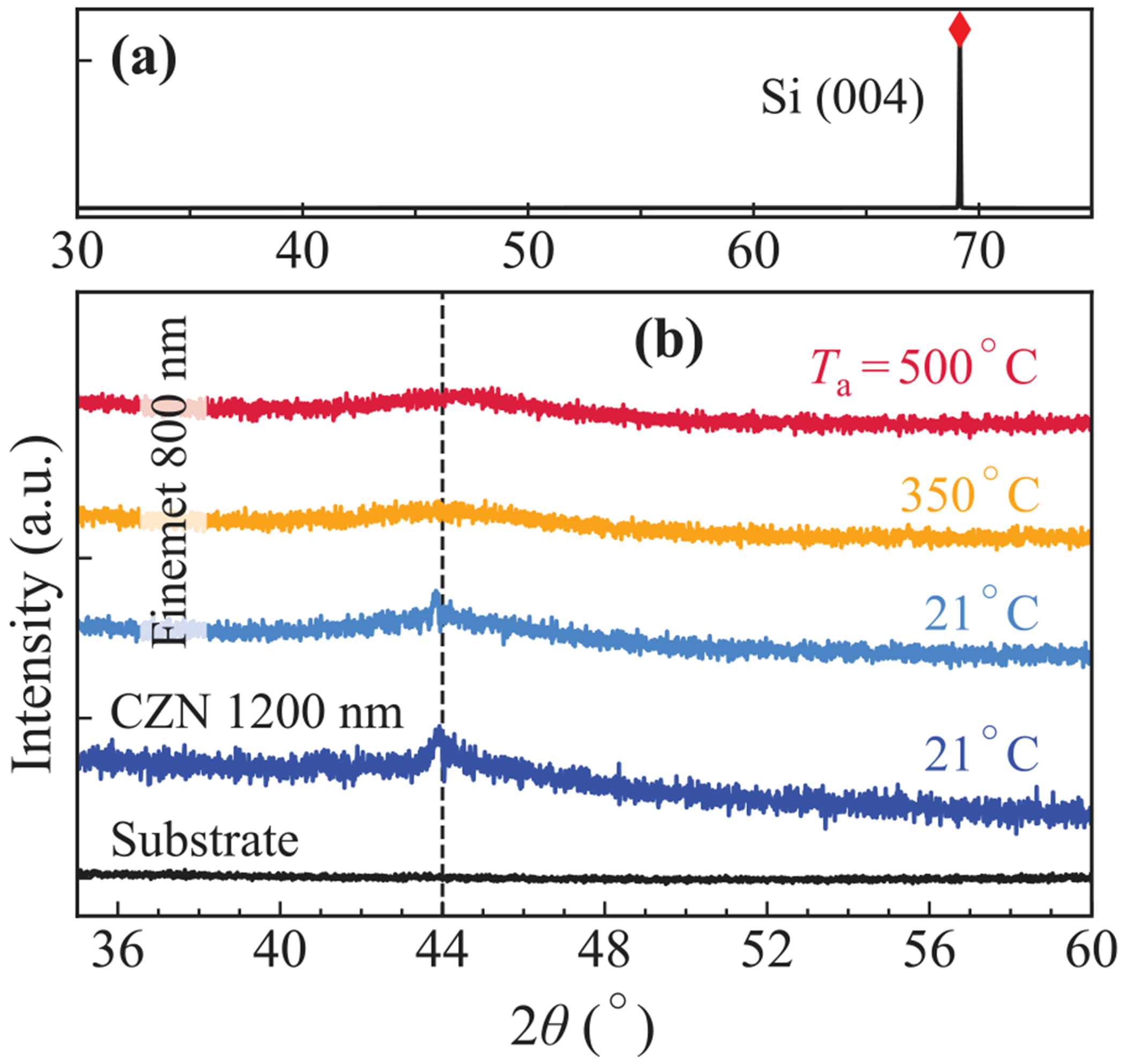
X-ray diffraction of CZN (1200 nm) and Finemet (800 nm) thin films at annealing conditions leading to their optimal magnetic softness. (a) Full scan indicates the prominent Si(004) peak at 2*θ* ≈ 69.13° from the substrate. (b) Magnified 35–60° window comparing the substrate, as-deposited CZN (21°C), and Finemet at 21°C, 350°C, and 500°C.

As shown in Figs. 1(a)–(b), *M*_r_ and *H*_c_ in CZN films both increase with annealing temperature. As-grown samples (measured at 21°C) exhibit the softest behaviour, with the thickest sample possessing the lowest *H*_c_ and *M*_r_. XRD provides structural confirmation of the as-deposited 1200 nm film in Fig. 2(b). The scan displays a broad amorphous peak near 2*θ* ≈ 44° and no Bragg peaks, confirming a fully amorphous room-temperature state. Upon annealing, however, both quantities gradually rise (more markedly in thicker samples), reaching a local maximum around 350°C.

**Fig. 3.**
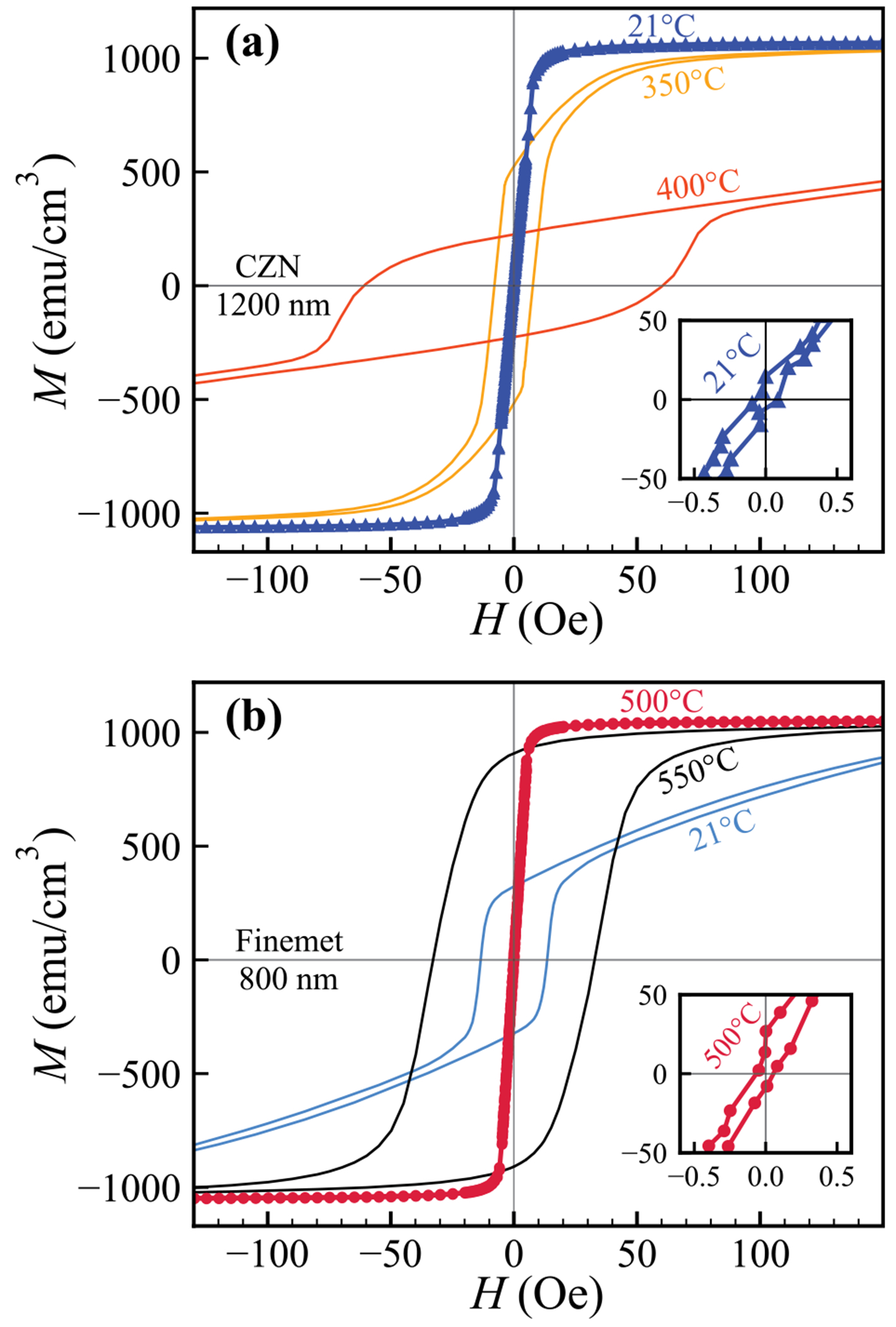
*M–H* hysteresis loops at different annealing temperatures for (a) CZN (1200 nm) and (b) Finemet (800 nm). Insets plots the low-field region, illustrating the minimised coercivity and remanence achieved under optimal annealing conditions.

This loss of extreme softness with heat treatment is evident in the *M–H* curves of 1200 nm CZN film (Fig. 3(a)). The as-deposited loop is ideal, with negligible coercivity and near-zero remanence, whereas after annealing at 350°C the loop broadens, and by 400°C it shows substantial *M*_r_ and *H*_c_ . Such changes signal the onset of a transition towards an ordered (partially crystalline or strain-relieved) phase in the Co-based film. At higher temperatures, this behaviour is consistent with the approach to the known crystallisation threshold in related Co-Zr-Nb amorphous thin films, beyond which rapid degradation of soft properties is reported [21,25–29].

The mechanism behind this behaviour can be understood via the random anisotropy framework [30,31]. In CZN’s fully amorphous state, the ferromagnetic exchange length is large compared to the structural correlation length, so exchange interactions effectively average out local magnetocrystalline anisotropies, yielding extremely low coercivity and near-zero hysteresis. Annealing-induced crystallisation suppresses this averaging. Once crystals form, the local magnetocrystalline anisotropy is no longer perfectly averaged and *H*_c_ increases. In addition, Co-based amorphous alloys often have non-zero magnetostriction, so the relief or imposition of internal stresses during annealing can introduce anisotropy [6]. Thus, the gradual loss of magnetic softness in CZN with temperature can be attributed to a combination of incipient crystallisation and stress-induced anisotropy. Both effects break the ideal random anisotropy condition of the as-deposited amorphous state and lead to increased domain-wall pinning.

In contrast, Finemet exhibits an almost opposite trend as displayed in Figs. 1(c)–(d). This material is engineered such that thermal treatment forms the *α*-Fe(Si) nanocrystals responsible for magnetic softness [17]. Upon annealing, *M*_r_ and *H*_c_ decrease sharply, reaching their minimum values at 500°C as the alloy transforms towards its nanocrystalline state [18]. XRD patterns of the 800 nm sample in Fig. 2(b) show a diffuse hump near 2*θ* ≈ 44°, characteristic of an amorphous structure. In our samples, the clearest evidence of nanocrystalline softening is therefore provided by the collapse of *H*_c_ and *M*_r_ in Figs. 1(c)–(d), which agrees with the established 450–500°C nanocrystallisation window for Finemet alloys [32–35].

Fig. 3(b) illustrates magnetic hysteresis loops for the 800 nm Finemet sample, highlighting this softening transformation. Initially, the *M–H* loop is relatively broad and square-shaped, indicating substantial coercivity and remanence. After annealing at 500°C, the loop collapses to a narrow shape with vanishing coercive field and a steep slope around zero field. This follows the random anisotropy model, where exchange coupling among ultrafine *α*-Fe(Si) grains averages local anisotropies and, together with stress relief, suppresses *H*_c_ [19]. Compositionally, Cu promotes dense nucleation of *α*-Fe(Si) crystallites, while Nb (together with B in the alloy) strongly inhibits coarsening, stabilising a ~ 10–15 nm grain size that underpins soft characteristics [17,36–38].

Annealing beyond the optimal temperature, however, reverses these gains in magnetic softness. When Finemet films are heated to 550°C, their hysteresis loop widens again and coercivity rises (Fig. 3(b)), indicating a deterioration in the soft magnetic state. This degradation at higher annealing temperatures is attributed to nanocrystal coarsening and the appearance of secondary phases [13,39]. Once the *α*-Fe(Si) grains grow beyond the ferromagnetic exchange length (~ 30–50 nm in these materials), the exchange averaging of anisotropy is no longer effective and significant magnetocrystalline anisotropy returns, leading to higher *H*_c_ [19,37]. Hence, a narrow thermal window (~ 450–550°C) yields the best balance of ultrafine grain size and stress relaxation in our films. Beyond this, anisotropy re-emerges and magnetic softness is compromised.

In summary, the combined magnetic and structural characterisation establishes complementary annealing pathways for these alloys. CZN attains its best properties in the fully amorphous as-deposited state, and degrades upon thermal treatment because of relaxation/ordering and crystallisation. Finemet requires a controlled annealing process at 500°C to enter its nanocrystalline regime in which exchange-averaged anisotropy and stress relief yield ultra-soft behaviour. Over-annealing of this material will drive coarsening and loss of soft properties.

### 3.2. Dependence of soft magnetic properties on film thickness

**Fig. 4.**
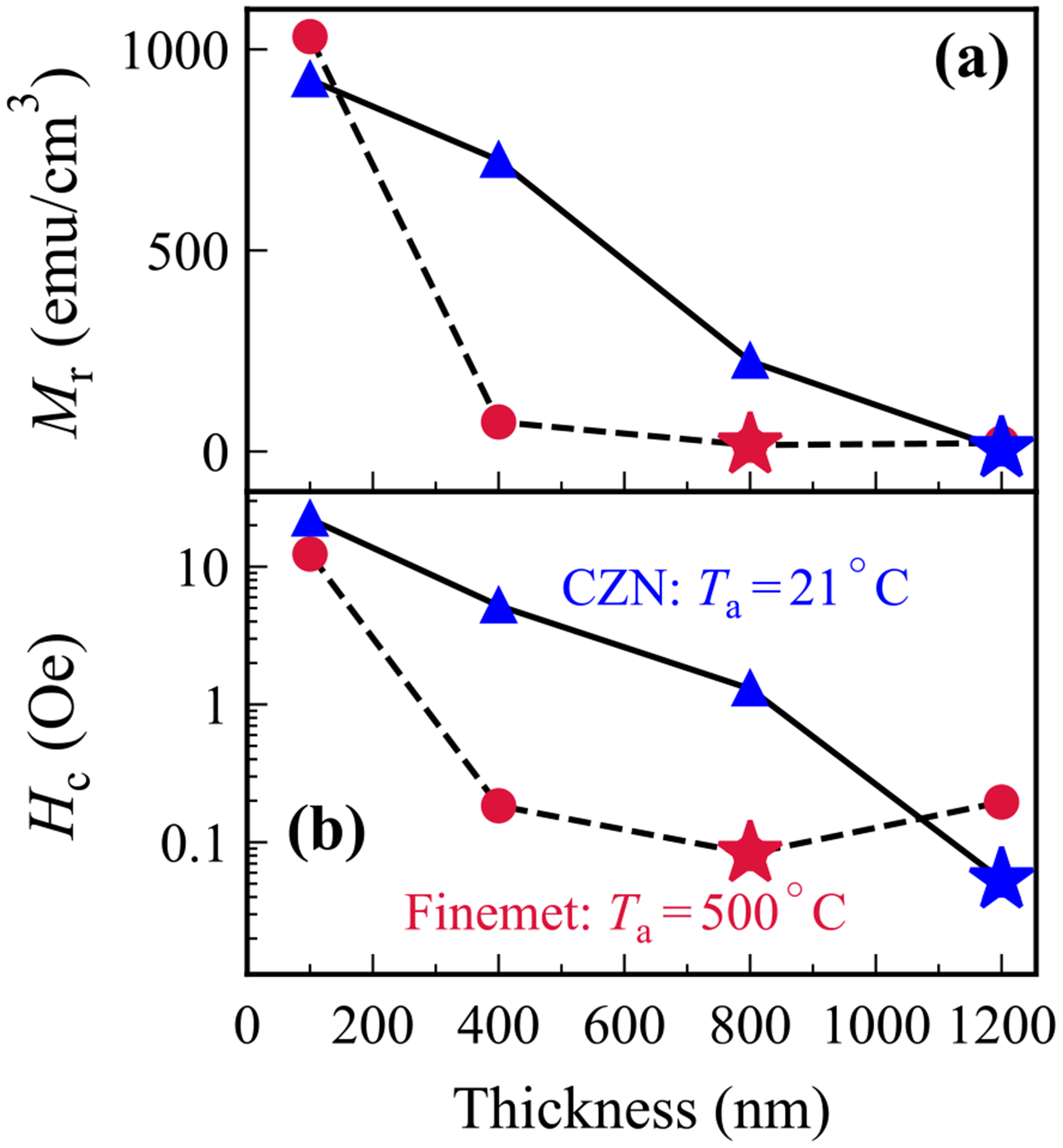
(a) Remanence magnetisation and (b) coercive field as a function of film thickness for (as-grown) CZN and Finemet (annealed at 500°C). Optimal magnetic softness is marked (*) for each material, emphasising their distinct trends with thickness.

**Fig. 5.**
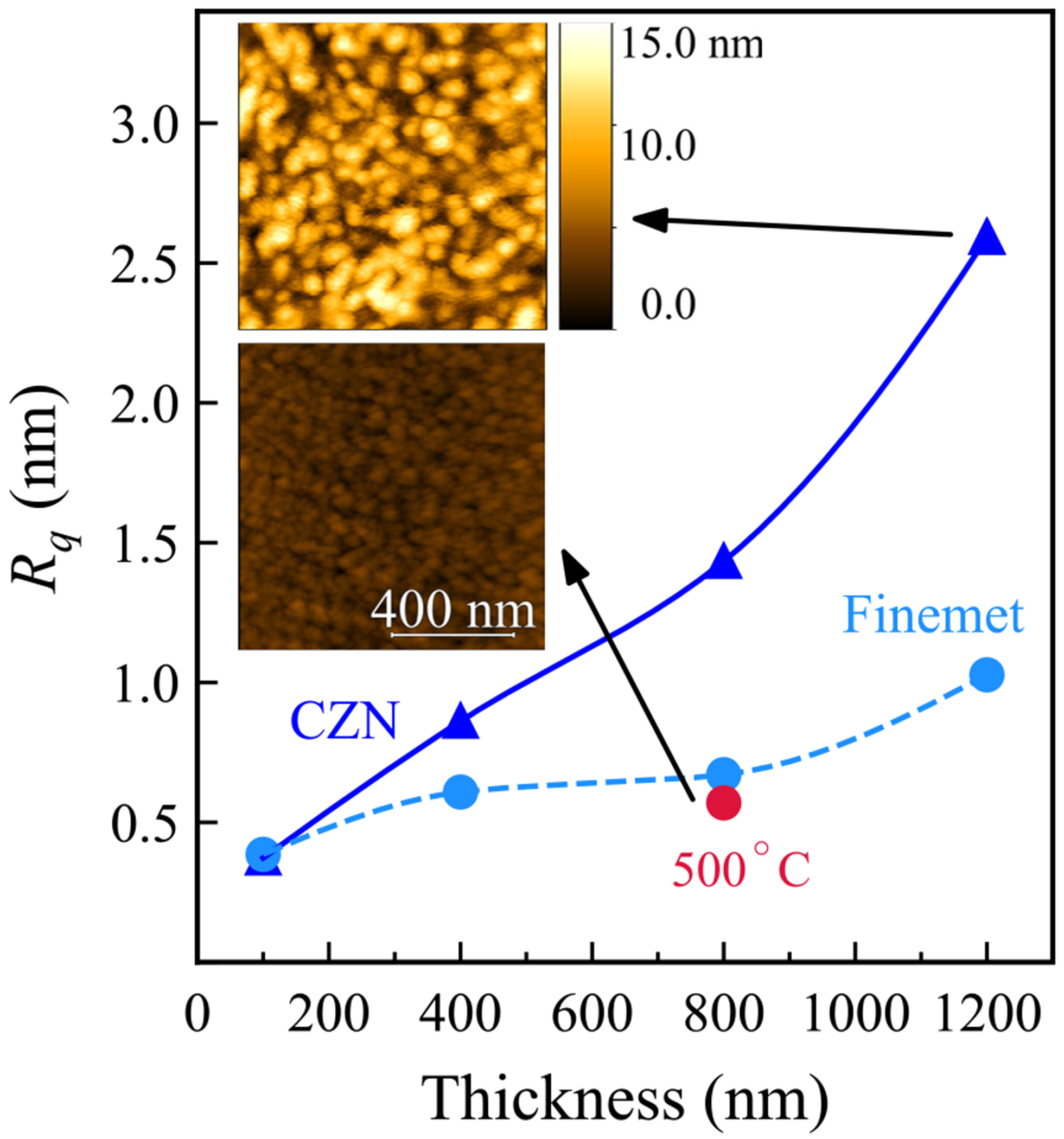
Root-mean-square (RMS) surface roughness (*R_q_*) versus thickness of as-grown CZN and Finemet. The red circle marks the data point for 800 nm Finemet film after annealing at 500°C. Insets are representative 1 μm^2^ AFM height maps for 1200 nm CZN and annealed 800 nm Finemet, both of which share the same colour scale shown on the top left micrograph.

Having identified the optimal annealing conditions for each material (room temperature for CZN and 500°C for Finemet), we now examine how film thickness affects their soft magnetic behaviour. Fig. 4 shows the variation of *M*_r_ and *H*_c_ with thickness for both materials, and Fig. 5 summarises the corresponding surface roughness trends.

As illustrated in Fig. 4(a), in CZN films, *M*_r_ and *H*_c_ both decrease as thickness increases. The thickest 1200 nm film exhibits the softest magnetic behaviour, with the lowest coercivity and remanence. This is in accordance with the expectation that thicker amorphous films, having a lower surface-to-volume ratio, present fewer interface pinning sites and reduced effective anisotropy, which facilitates easier magnetisation reversal [40]. Volume averaging also diminishes stress-induced anisotropy, further lowering *H*_c_. Similar thickness softening trends in Co-Zr-Nb alloys have been reported previously [41]. Our AFM data (Fig. 5) provide context: the 1200 nm film has root-mean-square (RMS) roughness *R_q_* = 2.59 nm, yet still yields the lowest *H*_c_. This indicates that intrinsic factors, namely domain configuration and reduced magnetoelastic anisotropy, dominate over any extrinsic pinning from surface asperities. While rougher surfaces can promote pinning [42] as in the case of thick CZN, the intrinsic softening with thickness clearly prevails.

Regarding Finemet, the thickness dependence trend is more complex. Fig. 4(b) demonstrates that *M*_r_ and *H*_c_ fall rapidly with thickness up to 800 nm, then *H*_c_ increases slightly by 1200 nm. The initial reduction is consistent with stress relaxation that reduces the magnetoelastic contribution to anisotropy [40]. At 800 nm, after annealing at 500°C, the film reaches its softest state with a well-developed nanocrystalline microstructure and minimal residual stress, as expected for optimally processed Finemet. AFM measurements at this condition give *R_q_* = 0.57 nm (Fig. 5), which is compatible with the very low *H*_c_. The modest rise in coercive force beyond 800 nm likely reflects the onset of additional extrinsic and microstructural contributions at larger thickness, such as slight surface roughening, grain growth, or the possible emergence of secondary phases, which can reintroduce pinning and anisotropy. Comparable non-monotonic thickness trends have been documented in related Finemet-type systems [40,41,43,44].

Overall, increasing film thickness improves magnetic softness in both CZN and Finemet alloys, but via different primary mechanisms. CZN’s coercivity reduction with thickness is driven by intrinsic effects associated with reduced demagnetising factor (larger volume) and suppressed magnetoelastic anisotropy. These intrinsic improvements dominate over roughness-related pinning in thick CZN films. Finemet, on the other hand, gains its thickness-induced softness largely from stress relaxation and nanostructural optimisation (especially after appropriate annealing). Once these gains are realised, further increasing the thickness can reintroduce minor losses in softness due to extrinsic factors (surface roughness or grain coarsening). Therefore, when designing magnetic flux concentrators or similar devices, one should choose film thickness based on the dominant effect for that material. For CZN, thicker is generally better to minimise coercivity, as long as the film remains amorphous and well-adhered. Whereas for Finemet, there is an optimal thickness after which the benefits taper off. These distinctions inform thickness selection for specific MFC applications, depending on processing constraints and targeted performance metrics.

## 4. Conclusions

**Fig. 6.**
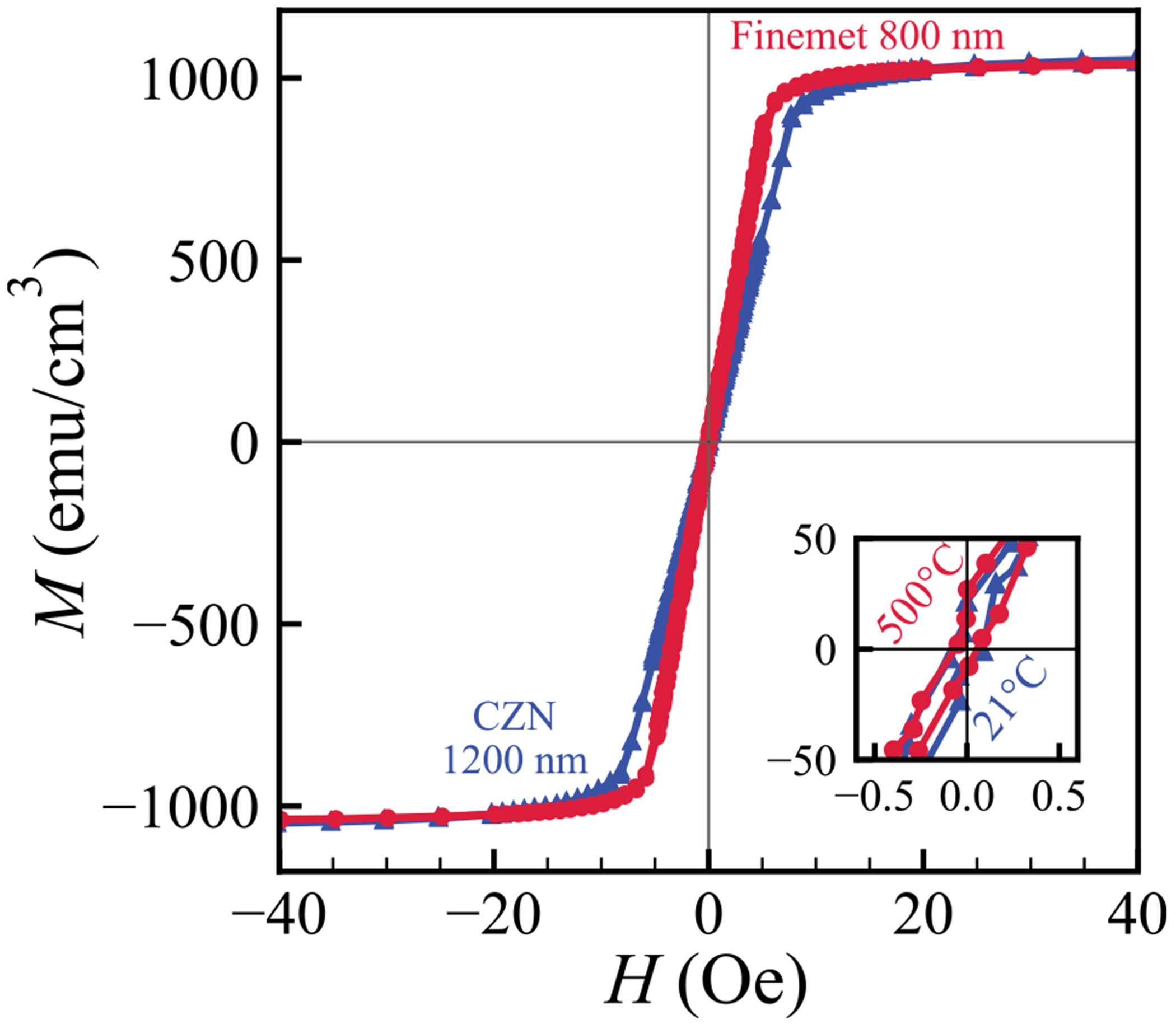
Comparison of *M–H* loops for the softest samples: CZN (1200 nm, as-grown) and Finemet (800 nm, 500°C annealed). Insets provide low-field detail, confirming near-zero coercivity in both cases.

We systematically compared CZN and Finemet samples across thickness and annealing conditions with the goal of minimising *H*_c_ and *M*_r_ in the regime relevant to thin-film, low-frequency magnetic flux concentrators. The softest samples are as-deposited CZN at 1200 nm, and Finemet at 800 nm following a 10-minute 500°C annealing procedure. Their quasi-static loops in Fig. 6 show near-zero coercivity, and the extracted quantities of interest in Table 1 summarise the practical advantage for device design.

Structural and morphological data support these outcomes. XRD confirms that the 1200 nm CZN film is fully amorphous at room temperature, in agreement with extreme softness in its as-grown state. AFM scans show high RMS roughness *R_q_* = 2.59 nm for this film, yet *H*_c_ remains the lowest among CZN samples. This indicates that the dominant improvements with thickness arise from intrinsic effects such as reduced magnetoelastic anisotropy and favourable domain configurations rather than surface pinning alone. For Finemet, XRD in conjunction with *M–H* loops point to the onset of *α*-Fe(Si) nanocrystallisation at 500°C, aligning with the collapse of *H*_c_ and an increase in *μ*. The optimised 800 nm Finemet shows a very smooth surface (*R_q_* = 0.57 nm), consistent with minimal extrinsic pinning and the observed ultra-soft response. The short 10-minute annealing dwell is sufficient to favour nucleation over growth, which promotes the formation of many sub-exchange-length *α*-Fe(Si) crystallites. This maximises random-anisotropy averaging and therefore yields soft magnetic behaviour. In practice, the short dwell limits thermal exposure while still delivering the desired nanocrystalline state.

Permeability and anisotropy further differentiate the two optima. CZN (1200 nm, as-deposited) attains a high permeability *μ* = 1611 with a modest differential anisotropy field ${Η}_{\text{k}}^{\text{diff}}=0.364$ Oe, which is compatible with low-field flux guiding and simple biasing schemes. Finemet (800 nm, 500°C, 10 minutes) reaches a higher permeability value *μ* = 2425 and an extremely small ${Η}_{\text{k}}^{\text{diff}}=0.003$ Oe, which implies a very weak in-plane anisotropy and highly reversible magnetisation. The larger *μ* and the near-vanishing ${Η}_{\text{k}}^{\text{diff}}$ favour stronger flux concentration with minimal rotational loss, while the short annealing time supports efficient processing.

These results lead to simple selection rules for thin-film MFCs. CZN is preferred when a no-anneal, low-temperature workflow is required and thicker films are acceptable, as increasing thickness strengthens softness while preserving CMOS/MEMS compatibility. Finemet is preferred when a brief 500°C heat treatment is permissible, since a 10-minute dwell at 800 nm produces the nanocrystalline state that gives higher permeability with very low anisotropy and coercivity. Hence, this material is best suited to pre-CMOS processing, off-chip concentrators, or substrates that can tolerate that step.

For flux concentrator applications, magnetic amplification depends on the film’s permeability and the concentrator’s geometry and aspect ratio, as shown in prior studies [2,3,45]. In this work, the 2 mm-diameter circular elements were patterned by photolithography solely to enable reproducible VSM measurements. Since thickness and annealing temperature trends arise from local microstructure, they are expected to transfer to larger-area films and patterned MFCs prepared under comparable conditions. In practical layouts, flux gain will combine the intrinsic softness reported here with the demagnetising factor set by shape and aspect ratio, which can be co-optimised during design.

This study focuses on quasi-static and low-frequency operation where *H*_c_ and *M*_r_ determine loss and reversibility, while *μ* and ${Η}_{\text{k}}^{\text{diff}}$), and low coercivity (*H*_c_ meaningfully characterise flux guiding capabilities. Within this regime, the pairing of magnetic metrics with XRD and AFM provides a clear, fabrication-ready pathway to select material, thickness, and annealing parameters for MFC integration.

## Figures and Tables

**Table 1:** Practical design summary table for MFC applications.

Material	Optimal thickness (nm)	*M*_s_ (emu/cm^3^ )	*H*_c_ (Oe)	*μ*	${Η}_{\text{k}}^{\text{diff}}$ (Oe)	Annealing	Use case
CZN	1200	1065	0.05	1611	0.364	None	MEMS, CMOS-compatible low-temperature sensors
Finemet	800	1055	0.08	2425	0.003	500^°^C	High-sensitivity MFCs where thermal budget allows

## Data Availability

The data that support the findings of this study are openly available in the Brown Digital Repository [46].
